# Supplementary material for: Auto/Paracrine C-Type Natriuretic Peptide/Cyclic GMP Signaling Prevents Endothelial Dysfunction
Source: Int J Mol Sci. 2024 Jul 16;25(14):7800. doi: 10.3390/ijms25147800 (PMC11277478; doi:10.3390/ijms25147800)
Supplement: Supplementary file 1 [file ijms-25-07800-s001.zip › ijms-3043750-supplementary.pdf]

Major Resources Table complementing the Materials and Methods Section

Animals

Species	Vendor or Source	Background Strain	Sex
Mice, Mus musculus	Animal facility of Würzburg University	Mixed C57BL6/J;129V	Males and Females

Genetic mouse lines

	Line	Vendor or Source	Genetic Background	Other Information
**Parent - Female**	GC-B$^{flox/flox}$	Kuhn lab	Mixed C57BL6J;129SV	first described in Špiranec et al. 2018
**Parent - Male**	Tie2Cre; GC-B$^{flox/flox}$	Kuhn lab	Mixed C57BL6J;129SV	first described in Špiranec et al. 2018
**Parent - male**	LDLR$^{-/-}$	Jax	B6.129S7-Ldlrtm1Her/J, Strain #:002207	-
**Parent - Female**	GC-B$^{flox/flox}$/ LDLR$^{-/-}$	Kuhn lab	Mixed C57BL6J;129SV	-
**Parent - Male**	Tie2Cre; GC-B$^{flox/flox}$/LDLR$^{-/-}$	Kuhn lab	Mixed C57BL6J;129SV	-

Primers and probes for quantitative RT-PCR

Target	Sequence of forward primer	Sequence of reverse primer	Universal Probe Library (Roche)
GC-B	5'-TGT TTG GTG TTT CCA GTT TCC-3'	5'-AGT TCT TCC CAG CGA ATG C-3'	Probe 67 (catalog 046 886 600 01)
P-Selectin	5'-CCG GAA AGA CTG GAT TGT TC-3'	5'-CCT GGA CAC TTG ATG GCT TC-3'	Probe 47 (catalog 046 880 740 01)
E-Selectin	5'-TCC TCT GGA GAG TGG AGT GC-3'	5'-GGT GGG TCA AAG CTT CAC AT-3'	Probe 19 (catalog 046 869 260 01)
VCAM-1	5'-TGG TGA AAT GGA ATC TGA ACC-3'	5'-CCC AGA TGG TGG TTT CCT T-3'	Probe 34 (catalog 046 876 710 01)
ET-1	5'-CTG CTG TTC GTG ACT TTC CA-3'	5'-TCT GCA CTC CAT TCT CAG CTC-3'	Probe 50 (catalog 046 881 120 01)
CNP	5'-AGC GGT CTG GGA TGT TAG TG-3'	5'-CGT TGG AGG TGT TTC CAG AT-3'	Probe 75 (cat. no. 046 889 880 01)
ß2-Microglobulin	5'-TAC GCC TGC AGA GTT AAG CA-3'	5'-GGT TCA AAT GAA TCT TCA GAG CA-3'	Probe 117 (catalog 046 935 150 01)
S12	5'-GAA GCT GCC AAA GCC TTA GA-3'	5'-AAC TGC AAC CAA CCA CCT TC-3'	No probe

The FastStart Essential Green Master (catalog no. 064 027 120 01, Roche) was used for S12. The FastStart Essential Probes Master (catalog no. 064 026 820 01) was used for all other targets.

Antibodies

Target antigen	Vendor or Source	Catalog #	Working concentration/dilution
α-actin (α-SMA)	Sigma-Aldrich	C6198	1:200
CD31	abcam, Berlin, Germany	ab124432	1 µg/ml
phosphorylated eNOS (Ser_{1177})	Cell Signaling, Leiden, Netherlands	#9571	1:1000
eNOS	BD Biosciences, Heidelberg, Germany	#610296	1:1000
Mac-2	Cedarlane, Burlington, Canada	CL8942AP	1:600
phosphorylated VASP (Ser_{239})	Cell Signaling, Leiden, Netherlands	#3114	1:1000
phosphorylated VASP (Ser_{157})	Cell Signaling, Leiden, Netherlands	#3111	1:1000
VASP	Cell Signaling, Leiden, Netherlands	#3112	1:1000
GAPDH	Cell Signaling, Leiden, Netherlands	#2118	1:10.000

Reference

Špiranec K, Chen W, Werner F, Nikolaev VO, Naruke T, Koch F, Werner A, Eder-Negrin P, Diéguez-Hurtado R, Adams RH, Baba HA, Schmidt H, Schuh K, Skryabin BV, Movahedi K, Schweda F, Kuhn M. Endothelial C-Type Natriuretic Peptide Acts on Pericytes to Regulate Microcirculatory Flow and Blood Pressure. *Circulation*. 2018;138:494-508

Supplementary Table S1

	Control females			EC GC-B KO females		
Cardiomyocyte area (µm²)	224.4	±	12.07	311.6	±	18.51*
CO (µl/min)	7507	±	1010	7757	±	673
SV (µl)	15.35	±	1.65	14.27	±	0.97
LV Pmax (mmHg)	104.9	±	4.1	105.1	±	2.2
LV Pmin (mmHg)	3.45	±	1.57	4.10	±	2.10
HR (beats/min)	487	±	29	543	±	26
EF (%)	54.6	±	2.5	49.5	±	3.5
dP/dtmax (mmHg/sec)	8593	±	873	8499	±	672
dP/dtmin (mmHg/sec)	-8089	±	545	-8252	±	801

Table S1. Left ventricular cardiomyocyte cross-sectional areas and contractile function of female control and EC GC-B KO mice. Morphometrical analyses of PAS-stained left ventricular sections (areas of 100-130 myocytes with central nucleus were determined in 3 sections per heart); LV contractile function was measured with invasive catheterization (Millar catheter) (n=6 per genotype; *$P<0.05$ versus controls; unpaired T-test). CO: cardiac output; SV: stroke volume; EF, ejection Fraction; HR, heart rate.

Supplementary Figure S1

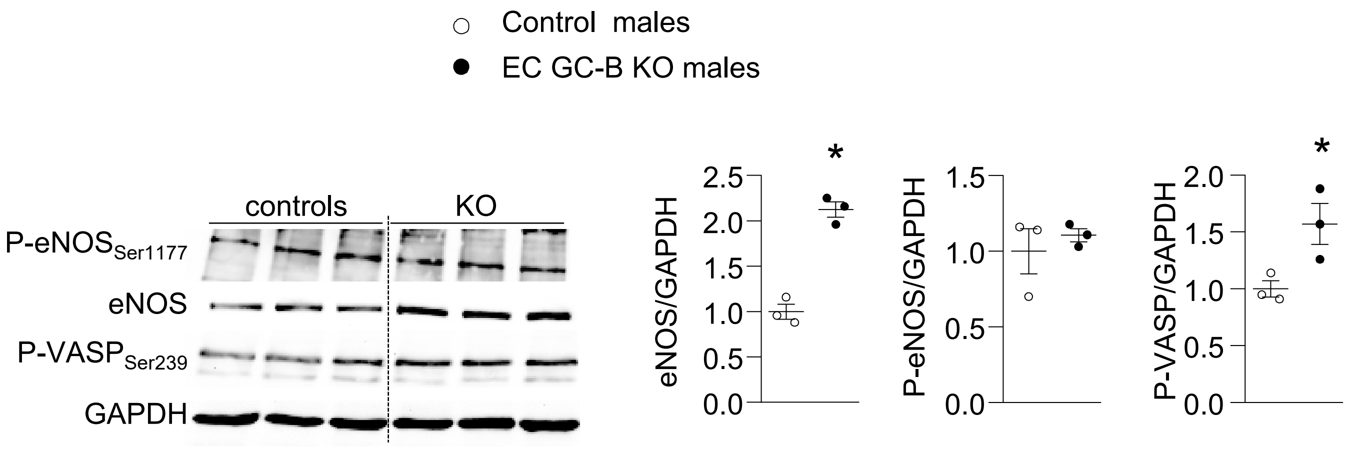

Figure S1. Enhanced eNOS expression and VASP-Serine$_{239}$ phosphorylation in aortae from male EC GC-B KO mice. Left: Immunoblots. Right: The expression/phosphorylation of target proteins was normalized to GAPDH and calculated as x-fold versus controls (n = 3; unpaired t-test; *P < 0.05 vs controls).

Supplementary Figure S2

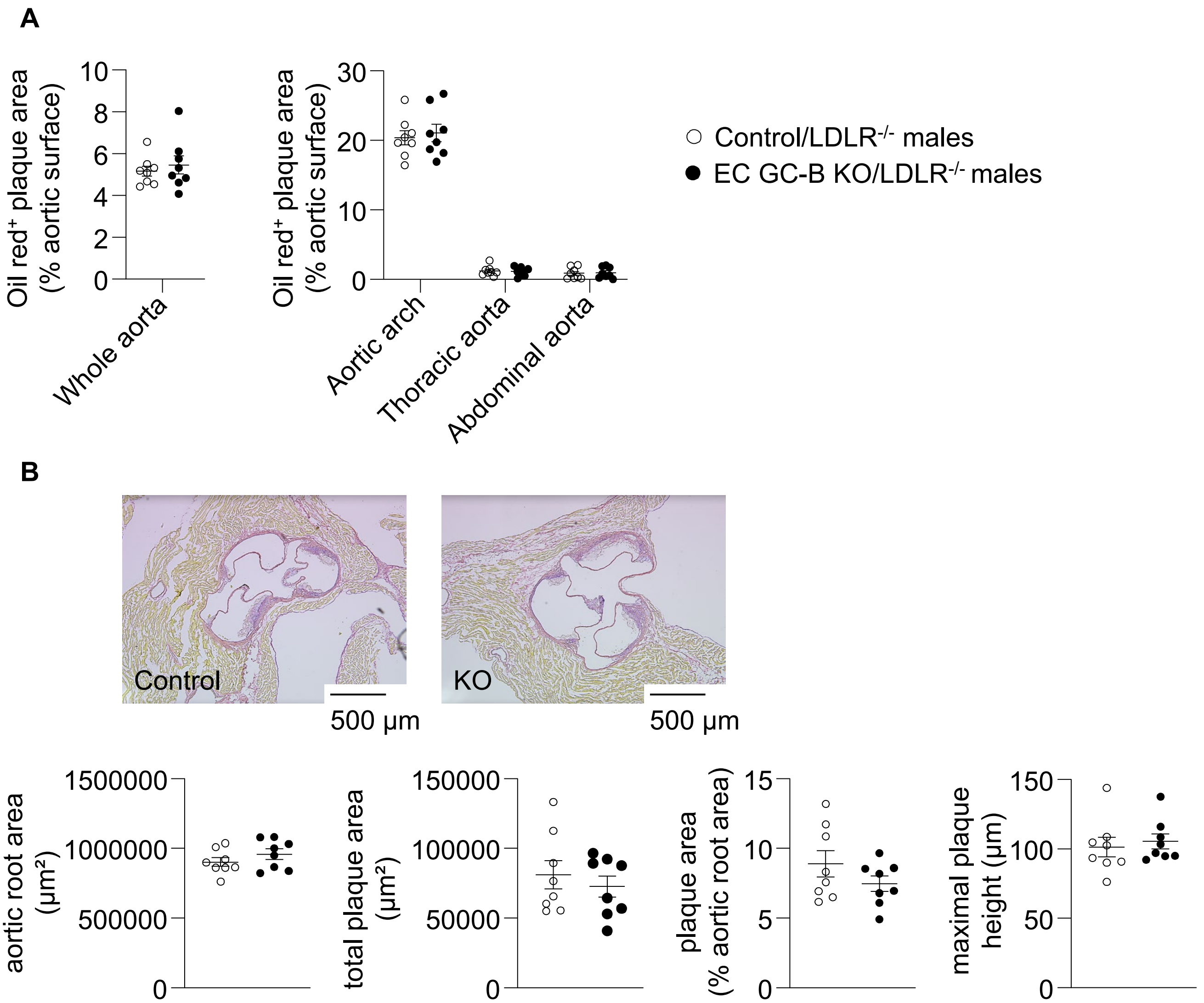

Figure S2. Deletion of endothelial GC-B did not impact atherosclerosis in male mice. A Oil red stainings of lipid depositions allowed quantifications throughout the whole aorta (left panel) and distinct aortic regions (right panel) (n= 8; unpaired t-test); **B.** Aldehyde fuchsin stainings of aortic root sections allowed to appreciate plaque areas and heights in this region (n = 8; unpaired t-test).
